# Usefulness of ^18^F-fluorodeoxyglucose positron emission tomography for T1 esophageal squamous cell carcinoma

**DOI:** 10.1097/MD.0000000000050879

**Published:** 2026-10-02

**Authors:** Dae Gon Ryu, Cheol Woong Choi, Seong Jang Kim, Su Jin Kim, Su Bum Park, Jin Ook Jang, Cheol Min Lee, Bong Soo Son

**Affiliations:** a Department of Internal Medicine, Pusan National University School of Medicine and Research Institute for Convergence of Biomedical Science and Technology, Pusan National University Yangsan Hospital, Yangsan, Korea; b Department of Nuclear Medicine, Pusan National University Yangsan Hospital, Yangsan, Korea; c Department of Thoracic and Cardiovascular Surgery, Pusan National University School of Medicine and Research Institute for Convergence of Biomedical Science and Technology, Pusan National University Yangsan Hospital, Yangsan, Korea.

**Keywords:** esophageal cancer, positron emission tomography, squamous cell carcinoma

## Abstract

The utility of ^18^F-fluorodeoxyglucose (FDG) positron emission tomography/computed tomography (PET/CT) for T1 esophageal cancer is unclear. This study analyzed the usefulness of FDG-PET for T1 esophageal squamous cell carcinoma (ESCC). Medical records of patients with T1 ESCC between December 2008 and July 2024 at Pusan National University Yangsan Hospital were retrospectively reviewed. The preoperative FDG-PET/CT findings and final pathological results of patients with T1 ESCC confirmed by surgery or endoscopic resection were compared. Among the 133 patients with T1 ESCC, 84 (63.2%) had mucosal cancer, 49 (36.8%) had submucosal cancer, and 9 (6.8%) had lymph node metastasis. The maximal standardized uptake value (SUVmax) of the PET/CT images was positively correlated with the invasion depth (*r* = 0.670; *P* < .001) and tumor length (*r* = 0.518; *P* < .001). PET confirmed FDG uptake in 8 cases of mucosal cancer and 35 cases of submucosal cancer (9.5% vs 71.4%; *P* < .001). The negative predictive value and positive predictive value of FDG-PET/CT for identifying mucosal cancer were 84% and 81%, respectively. Of the 8 mucosal cancers in which FDG uptake was observed during PET, 5 (62.5%) were whole or near-circumferential lesions. FDG uptake observed using PET/CT was positively correlated with the invasion depth and tumor length of T1 ESCC. FDG-PET/CT may provide complementary information for risk stratification in patients with T1 ESCC.

## 1. Introduction

Esophageal cancer occurs frequently in older individuals, and its prevalence increases as life expectancy increases.^[1,2]^ The main histological types of esophageal cancer are squamous cell carcinoma (SCC) and adenocarcinoma.^[2]^ SCC is the most common histological type worldwide, and it is overwhelmingly prevalent in East Asia, including South Korea.^[2,3]^ Endoscopic screening programs for gastric cancer are available for the general population in Korea and Japan; therefore, more T1 esophageal cancer cases are being discovered early.^[4,5]^ The treatment of T1 esophageal squamous cell carcinoma (ESCC) includes endoscopic resection for mucosal cancer and surgery for submucosal cancer.^[6-8]^ However, caution is required for muscularis mucosal (M3) cancer because it has the potential to metastasize to the lymph nodes.^[7-10]^ Esophageal cancer treatment guidelines recommend surgery rather than endoscopic resection for whole circumferential M3 cancer because the possibility of lymph node metastasis and intractable strictures after endoscopic resection can be problematic.^[7,11]^

^18^F-fluorodeoxyglucose (FDG) positron emission tomography (PET)/computed tomography (CT) has been successfully used to diagnose, stage, assess the treatment response, and determine the prognosis of various cancers. FDG-PET/CT is also widely used to treat gastrointestinal cancers.^[12]^ However, the utility of FDG-PET/CT for T1 esophageal cancer is unclear. Compared to other cancers, gastrointestinal cancers are often detected during an early stage. Early-stage gastrointestinal cancers are small and have a shallow depth; therefore, FDG uptake is often not observed using PET.^[12,13]^ Additionally, these cancers often cause confusion because of physiological FDG uptake in the gastrointestinal tract.^[12,14]^ In this study, we aimed to identify the role of FDG-PET/CT in the treatment of T1 ESCC.

## 2. Materials and methods

### 2.1. Patients

The medical records of patients with esophageal cancer treated between December 2008 and July 2024 at Pusan National University Yangsan Hospital in South Korea were reviewed. Patients with histologically confirmed T1 ESCC who underwent surgery or endoscopic resection were included. Patients who did not undergo FDG-PET/CT before treatment, those with multiple lesions, and those who were not followed up after treatment were excluded. All patients included in this study underwent upper gastrointestinal endoscopy, chest/abdominal CT, and FDG-PET/CT before treatment. Although endoscopic ultrasound (EUS) was performed before treatment whenever the invasion depth was uncertain and the findings could influence treatment selection, it was not performed in some patients who were uncooperative, those whose preoperative diagnosis was not cancer, those with whole circumferential lesions, or those with obvious submucosal invasive cancer on conventional endoscopy. Based on the findings of endoscopy, CT, EUS, and FDG-PET/CT, surgery was recommended for patients with suspected submucosal invasive cancer or whole circumferential lesions. Endoscopic resection was primarily performed for lesions considered to be confined to the mucosa without evidence of lymph node metastasis. Definitive chemoradiotherapy was recommended for patients with cervical esophageal cancer, even when the lesion was clinically staged as T1.

This study was approved by the Ethics Committee of Pusan National University Yangsan Hospital review board (Institutional Review Board no. 55-2024-107), which waived the requirement for informed consent because medical records were anonymized before analysis. All methods were performed in accordance with the relevant guidelines and regulations by the Ethics Committee of Pusan National University Yangsan Hospital review board.

### 2.2. Endoscopy and endoscopic ultrasound

The location, size, and macroscopic type of all cancers were recorded at the time of endoscopy. The tumor location and macroscopic type were classified according to the Japanese Classification of Esophageal Cancer.^[15]^ All lesions were identified by narrow-band imaging and Lugol’s iodine chromoendoscopy. A whole circumferential lesion was defined as cancer surrounding the entire esophageal lumen, and a near circumferential lesion was defined as cancer surrounding more than 3-quarters of the esophageal lumen. EUS was performed by one of 3 endoscopists with more than 7 years of EUS experience. All examinations were performed under conscious sedation via intravenous midazolam (2.5–8 mg). The invasion depth was measured using an EUS catheter probe (20 MHz; Olympus, Tokyo, Japan) with the water-filled method, and the lymph nodes were evaluated using a radial EUS scope (GF-UE 160-AL5 Radial Array Ultrasound Gastrovideoscope; Olympus). Lymph nodes were considered malignant when they were larger than 10 mm on any axis, hypoechoic, rounded, and had clearly defined margins.^[16]^

### 2.3. 18F-fluorodeoxyglucose-positron emission tomography/computed tomography

FDG-PET/CT was performed within 4 weeks before treatment. PET/CT was performed using a Gemini PET/CT scanner (Philips, Milpitas). Patients were instructed to fast for 6 hours. Blood glucose was measured to ensure a level lower than 150 mg/dL prior to the injection of 370 to 444 MBq ^18^F-FDG. Simultaneous emission–transmission PET scans were performed 1 hour after the FDG injection. The FDG-PET/CT acquisition protocol included an initial CT evaluation (140 kV; 80 mA; helical slices, 2; 0.5 seconds per rotation; pitch, 6:1; slice thickness, 5 mm), followed by FDG-PET acquisition in the 2-dimensional mode for 4 minutes per field of view. Images were reconstructed using an iterative median root prior to the reconstruction algorithm. Trans-axial, coronal, and sagittal image sets were displayed on the monitor of a high-resolution computer as linear grayscale. During the semiquantitative analysis, the regions of interest were selected semiautomatically as the most intense areas of FDG accumulation in the primary tumor on the PET image, and the maximal standardized uptake value (SUVmax) was calculated according to the following formula: PET count of the most intense point × calibration factor (MBq/kg)/injection dose (MBq)/body weight (kg). The FDG-PET/CT datasets were independently reviewed by a single board-certified nuclear medicine specialist with 30 years of experience in PET/CT interpretation. Tumors with an SUVmax ≥2.5 were considered to demonstrate positive FDG uptake.^[17]^ Accordingly, cases with an SUVmax ≥2.5 in the primary tumor or lymph nodes were considered PET-positive.

### 2.4. Surgery

Patients selected to undergo surgery underwent Ivor Lewis esophagectomy with an intrathoracic anastomosis and 2-field or 3-field lymph node dissection if anatomical abnormalities were not present. Patients with anatomical abnormalities, such as those who have undergone gastrectomy, underwent jejunal or colonic interposition. Anastomotic leakage was assessed 1 week after surgery using esophagography and endoscopy. Postoperative mortality was defined as that within 30 days after surgery or during postoperative hospitalization. A pathological analysis was performed to evaluate resection completeness (R0) and lymph node metastasis.

### 2.5. Endoscopic resection

T1 esophageal cancer cases without evidence of lymph node metastasis during the preoperative examination and those localized to the mucosa on EUS were treated with endoscopic resection. When submucosal invasive cancer was suspected, the patients underwent endoscopic resection because of the high surgical risk. Most patients underwent endoscopic submucosal dissection (ESD), and most procedures were conducted under conscious sedation via intravenous midazolam without general anesthesia. If no complications occurred, the patient was discharged 2 days after the procedure. Patients who underwent endoscopic resection were followed every 6 months with upper gastrointestinal endoscopy, chest and abdominal CT, and laboratory tests according to our institutional surveillance protocol. FDG-PET/CT was performed only when recurrence was clinically suspected.

### 2.6. Histopathological evaluation

Surgically resected primary tumors and lymph nodes were sliced at 4-mm intervals, and endoscopically resected primary tumors were sliced at 2-mm intervals. Two pathologists with more than 5 years of experience independently assessed specimens of primary tumors and lymph nodes to determine the tumor invasion depth, macroscopic type, lymphovascular invasion status, and nodal metastasis. The specimens were pathologically reviewed according to the Japanese Classification of Esophageal Cancer (11th edition).^[15]^ Infiltration of esophageal cancers was subclassified into the following 6 categories: M1, intraepithelial carcinoma; M2, tumor infiltrating the lamina propria; M3, tumor infiltrating the muscularis mucosa; SM1, tumor invasion to the shallow strata (medial third) of the submucosal layer or≤200 μm from the muscularis mucosae in endoscopically resected specimens; SM2, tumor invasion into the middle strata (middle third) of the submucosal layer; and SM3, massive tumor invasion into the deep strata (outer third) of the submucosal layer.

### 2.7. Statistical analysis

Spearman rank correlation was used to determine the correlation between the SUVmax and tumor invasion depth. The Pearson correlation coefficient was used to determine the correlation between the SUVmax and tumor length. Receiver-operating characteristic (ROC) curve analysis was performed, and the area under the curve (AUC) with its 95% confidence interval (CI) was calculated for each imaging modality, as appropriate. For the interpretation of diagnostic performance, an AUC ≥ 0.90 was considered to indicate excellent diagnostic performance.^[18]^ The sensitivity, specificity, positive predictive value (PPV), negative predictive value (NPV), and accuracy, as well as the associated 95% CIs of the diagnostic tests of submucosal invasive cancer and lymph node metastases, were evaluated. Statistical significance was set at *P* < .05. Statistical Package for the Social Sciences version 27.0 (IBM Corp, Armonk) was used for all statistical analyses.

## 3. Results

### 3.1. Baseline characteristics in patients

During the study period, 152 patients received a histological diagnosis of T1 ESCC confirmed by surgery or endoscopic resection. Of these patients, 133 were analyzed after excluding 12 patients who did not undergo PET, 5 patients who had multiple lesions, and 2 patients who were not followed up after treatment. The mean age of the patients was 67 years (range, 42–85 years), and 118 (88.7%) patients were male. Eighty (60.2%) patients underwent surgery, and 53 (39.8%) patients underwent endoscopic resection. Lymph node metastasis was surgically confirmed in 9 (6.8%) patients. There were 84 (63.2%) cases of mucosal cancer and 49 (36.8%) cases of submucosal cancer. The baseline characteristics of the patients are summarized in Table 1. The most common tumor location was the midthoracic region (66.2%). Most mucosal cancers were type IIb (92.9%), and most submucosal cancers were elevated (type I, 14.3%; type IIa, 49.0%). The tumor length of submucosal cancers was greater than that of mucosal cancers (median 1.6 cm vs 3.4 cm; *P* < .001). Lymph node metastases were more commonly observed with submucosal cancers than they were with mucosal cancers (2.4% vs 14.3%; *P* = .020).

**Table 1:** Baseline characteristics of patients.

	Mucosal cancer (n = 84)	Submucosal cancer (n = 49)	*P* value
Age, median (range) yr	67 (42–85)	67 (42–82)	.626
Male, n (%)	72 (85.7)	46 (93.9)	.163
Location, n (%)			
Cervical	0 (0)	1 (2.0)	.314
Upper thoracic	3 (3.6)	8 (16.3)	.018
Mid thoracic	54 (64.3)	34 (69.4)	.549
Lower thoracic	27 (32.1)	6 (12.2)	.013
Macroscopic type, n (%)			
I	2 (2.4)	7 (14.3)	.020
IIa	4 (4.8)	24 (49.0)	<.001
IIb	78 (92.9)	8 (16.3)	<.001
IIc	0 (0)	7 (14.3)	.021
III	0 (0)	3 (6.1)	.095
Whole-circumferential, n (%)	5 (6.0)	8 (16.3)	.061
Treatment, n (%)			
Surgery	38 (45.2)	42 (85.7)	<.001
Endoscopic resection	46 (54.8)	7 (14.3)	
Length of tumor, median (range) cm	1.6 (0.1–8.5)	3.4 (0.6–10.0)	<.001
Lympho-vascular invasion, n (%)	2 (2.4)	7 (14.3)	.020
Lymph node metastasis, n (%)	2 (2.4)	7 (14.3)	.020

### 3.2. FDG uptake by the primary tumor

Of the 133 patients, 43 (32.3%) exhibited FDG uptake at the primary tumor site. FDG uptake was observed in 8 (9.5%) of 84 and 35 (71.4%) of 49 patients with mucosal cancer and submucosal cancer, respectively. The invasion depth was positively correlated with the SUVmax of the primary tumor (*r* = 0.670; *P* < .001; Table 2 and Fig. 1A). The median SUVmax of mucosal cancers was 0 (range, 0–5.8), and that of submucosal cancers was 5.0 (range, 0–17.5). The tumor length was also positively correlated with the SUVmax of the primary tumor (*r* = 0.518, *P* < .001; Table 3 and Fig. 1B). When the tumor length was ≥3 cm, FDG uptake was confirmed in 28 (71.8%) of 39 cancer cases. Even when the invasion depth and tumor length were controlled for and a partial correlation analysis was performed, they remained significantly correlated with the SUVmax (invasion depth: *r* = 0.600, *P* < .001; tumor length: *r* = 0.393, *P* < .001). Of the 8 mucosal cancers in which FDG uptake was observed, 5 (62.5%) were whole or near-circumferential lesions.

**Table 2:** Relation between FDG uptake by primary tumor and invasion depth.

Depth of invasion	Number	Positive uptake, n (%)	Median SUVmax (IQR, range)	*P* value
M1	21	0 (0)	0 (0–0, 0–0)	<.001
M2	39	5 (12.8)	0 (0–0, 0–5.8)	
M3	24	3 (12.5)	0 (0–0, 0–3.8)	
M	84	8 (9.5)	0 (0–0, 0–5.8)	
SM1	6	0 (0)	0 (0–0, 0–0)	
SM2	12	8 (66.7)	3.8 (0–6.0, 0–11.2)	
SM3	31	27 (90.3)	6.5 (4.5–9.6, 0–17.5)	
SM	49	35 (71.4)	5.0 (0–8.0, 0–17.5)	
Total	133	43 (32.3)	0 (0–4.2, 0–17.5)	

FDG = ^18^F-fluorodeoxyglucose, IQR = interquartile range, M1 = intraepithelial carcinoma, M2 = tumor infiltrating the lamina propria, M3 = tumor infiltrating the muscularis mucosa, SM1 = tumor invasion to the shallow strata (medial third) of the submucosal layer or ≤200 μm from the muscularis mucosae in endoscopically resected specimens, SM2 = tumor invasion into the middle strata (middle third) of the submucosal layer, SM3 = massive tumor invasion into the deep strata (outer third) of the submucosal layer, SUVmax = maximal standardized uptake value.

**Table 3:** Relation between FDG uptake by primary tumor and tumor length.

Tumor length (cm)	Number	Positive uptake, n (%)	Median SUVmax (IQR, range)	*P* value
<1.0	24	0 (0)	0 (0–0, 0–0)	*< 0.001*
1.0–2.0	42	5 (11.9)	0 (0–2.7, 0–6.5)	
2.0–3.0	25	9 (36.0)	0 (0–5.0, 0–14.3)	
3.0–4.0	14	9 (64.3)	5.4 (0–7.9, 0–17.5)	
4.0–5.0	12	9 (69.2)	4.7 (0–7.1, 0–15.8)	
≥5.0	16	8 (72.7)	4.8 (0–7.1, 0–10.4)	
Total	133	43 (32.3)	0 (0–4.2, 0–17.5)	

FDG = ^18^F-fluorodeoxyglucose, IQR = interquartile range, SUVmax = maximal standardized uptake value.

**Figure 1.**
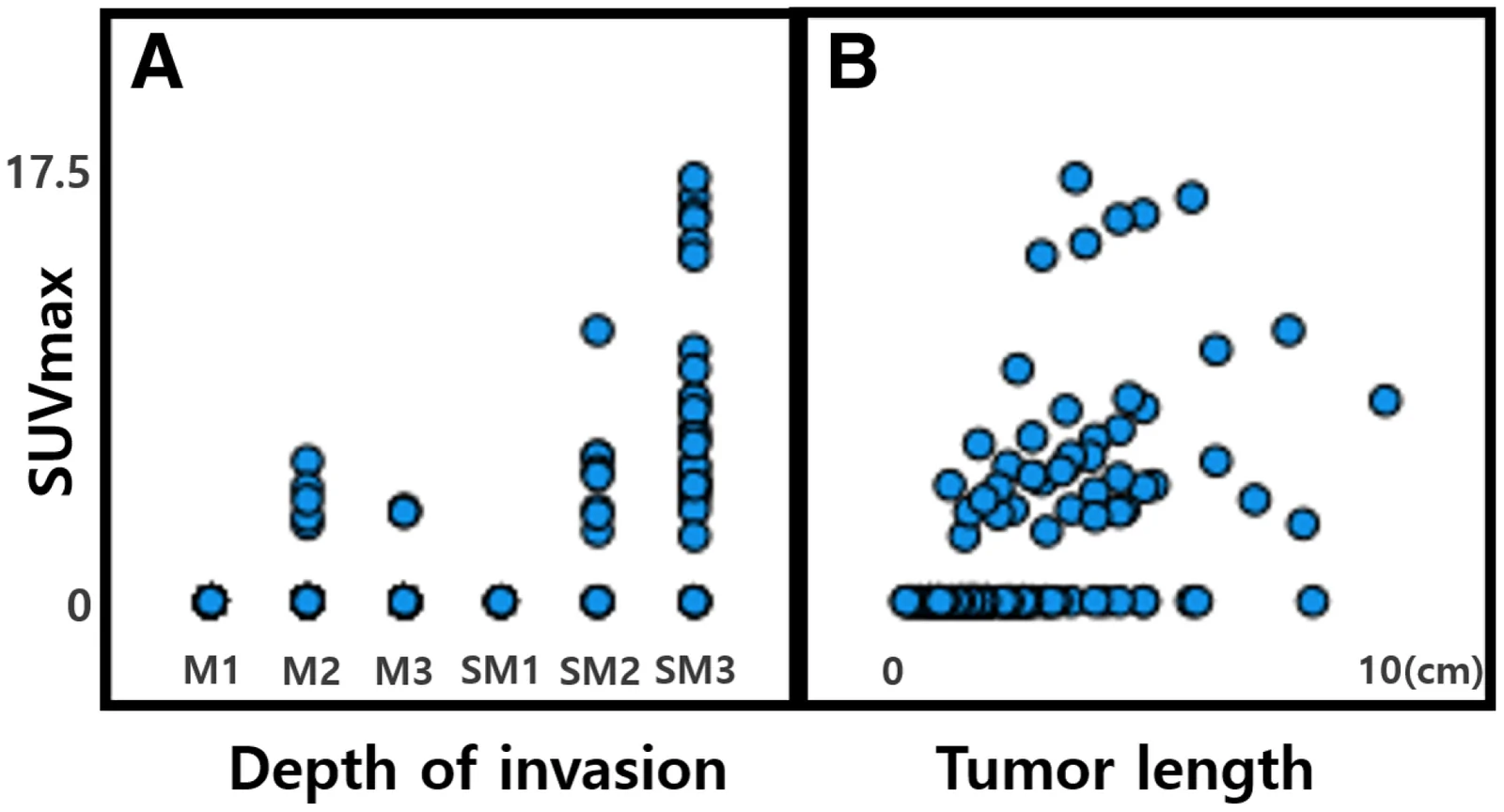
Scatterplot showing the correlation of SUVmax with invasion depth and tumor length. (A) Scatterplot of invasion depth and SUVmax. (B) Scatterplot of tumor length and SUVmax. M1 = intraepithelial carcinoma, M2 = tumor infiltrating the lamina propria, M3 = tumor infiltrating the muscularis mucosa, SM1 = tumor invasion to the shallow strata (medial third) of the submucosal layer or ≤200 μm from the muscularis mucosae in endoscopically resected specimens, SM2 = tumor invasion into the middle strata (middle third) of the submucosal layer, SM3 = massive tumor invasion into the deep strata (outer third) of the submucosal layer, SUVmax = maximal standardized uptake value.

### 3.3. Comparison of FDG-PET/CT and EUS for predicting submucosal cancer

The sensitivity, specificity, positive predictive value (PPV), negative predictive value (NPV), and accuracy of FDG-PET/CT for diagnosing submucosal cancer were 0.71, 0.90, 0.81, 0.84, and 0.83, respectively. EUS was performed for 108 (81.2%) patients, and the sensitivity, specificity, PPV, NPV, and accuracy for diagnosing submucosal cancer were 0.71, 0.71, 0.59, 0.81, and 0.71, respectively. The area under the curve values of FDG-PET/CT and EUS for the diagnosis of submucosal cancer were 0.84 and 0.73, respectively (Table 4). The negative predictive value and positive predictive value of FDG-PET/CT for identifying mucosal cancer were 0.84 and 0.81, respectively. The sensitivity and specificity of PET for deep submucosal (SM2 and SM3) cancer were 0.57 and 0.91, respectively.

**Table 4:** Diagnostic values of FDG-PET/CT and EUS for predicting submucosal cancer.

	FDG-PET/CT (n = 133)	EUS (n = 108)
Sensitivity (95% CI)	0.71 (0.57–0.83)	0.71 (0.54–0.85)
Specificity (95% CI)	0.90 (0.82–0.96)	0.71 (0.59–0.82)
PPV (95% CI)	0.81 (0.69–0.90)	0.59 (0.48–0.69)
NPV (95% CI)	0.84 (0.78–0.89)	0.81 (0.72–0.88)
Accuracy (95% CI)	0.83 (0.76–0.89)	0.71 (0.61–0.80)
AUC (95% CI)	0.84 (0.73–0.89)	0.73 (0.60–0.80)

AUC = area under the curve, CI = confidence intervals, EUS = endoscopic ultrasound, FDG = ^18^F-fluorodeoxyglucose, NPV = negative predictive value, PET/CT = positron emission tomography/computed tomography, PPV = positive predictive value.

### 3.4. FDG uptake by metastatic lymph node

Nine patients had surgically confirmed lymph node metastasis. Patients who underwent endoscopic resection had no evidence of lymph node metastasis on pretreatment staging and were followed for a median of 36 months (range, 2–176 months). Among patients with lymph node metastasis, 4 had FDG uptake in the lymph nodes. The SUVmax of lymph nodes in these patients ranged from 4.7 to 11.4. FDG uptake was observed in the lymph nodes, but not in the primary tumor in 1 patient. Two patients exhibited uptake in the periesophageal lymph nodes, with SUVmax values of 2.8 and 4.1, respectively, but lymph node metastasis was not observed.

### 3.5. Comparison of FDG-PET/CT with EUS and CT for the prediction of lymph node metastasis

The sensitivity, specificity, PPV, NPV, and accuracy of FDG-PET/CT for the diagnosis of lymph node metastasis were 0.44, 0.98, 0.67, 0.96, and 0.95, respectively. The sensitivity, specificity, PPV, NPV, and accuracy of EUS were 0.29, 0.97, 0.40, 0.95, and 0.93, respectively. The sensitivity, specificity, PPV, NPV, and accuracy of CT were 0.22, 0.96, 0.29, 0.94, and 0.91, respectively (Table 5). When PET positivity was defined as FDG uptake in either the primary tumor or regional lymph nodes, the sensitivity, specificity, PPV, NPV, and accuracy for predicting lymph node metastasis were 0.78, 0.69, 0.16, 0.98, and 0.70, respectively.

**Table 5:** Diagnostic values of FDG-PET/CT, EUS, and CT for predicting lymph node metastasis.

	FDG-PET/CT	EUS	CT
Sensitivity (95% CI)	0.44 (0.14–0.79)	0.29 (0.04–0.71)	0.22 (0.03–0.60)
Specificity (95% CI)	0.98 (0.94–1.00)	0.97 (0.92–0.99)	0.96 (0.91–0.99)
PPV (95% CI)	0.67 (0.30–0.90)	0.40 (0.12–0.97)	0.29 (0.08–0.64)
NPV (95% CI)	0.96 (0.93–0.98)	0.95 (0.92–0.97)	0.94 (0.92–0.96)
Accuracy (95% CI)	0.95 (0.89–0.98)	0.93 (0.86–0.97)	0.91 (0.84–0.95)

AUC = area under the curve, CI = confidence intervals, EUS = endoscopic ultrasound, FDG = ^18^F-fluorodeoxyglucose, NPV = negative predictive value, PET/CT = positron emission tomography/computed tomography, PPV = positive predictive value.

### 3.6. Incidental findings of other malignancies using FDG-PET/CT

Malignancies other than esophageal cancer were incidentally discovered by FDG-PET/CT in 3 (2.3%) patients. One patient underwent surgery for bile duct cancer (pTisN0M0, intraductal papillary neoplasm of the bile duct associated with invasive carcinoma) at 1 month after esophageal ESD, and the other 2 patients underwent surgery for colon cancer (pT2N0M0, adenocarcinoma) and lung cancer (pT2aN0M0, adenocarcinoma) at 2 months after esophagectomy. All patients are currently undergoing follow-up without any specific additional treatment.

## 4. Discussion

In our study, the negative predictive value and positive predictive value of FDG-PET/CT for identifying mucosal cancer were 84% and 81%, respectively. Additionally, FDG uptake was positively correlated with tumor length, and 62.5% of patients with mucosal cancer and positive PET results had whole or near circumferential cancer. The role of FDG-PET/CT in early-stage cancer is minimal, especially in gastrointestinal cancers, which are often detected at an early stage using screening endoscopy. However, the results of our study suggest that FDG-PET/CT may provide complementary information for risk stratification in patients with T1 ESCC and may assist in the overall clinical assessment when interpreted together with conventional endoscopic findings. PET findings alone, however, should not determine the treatment strategy.

FDG-PET/CT has been used to diagnose, stage, assess treatment response, detect recurrence, and determine the prognosis of various cancers, including esophageal cancer. All esophageal cancer guidelines mention the need for FDG-PET/CT for staging.^[6-8]^ The National Comprehensive Cancer Network guidelines recommend FDG-PET/CT for patients without distant metastasis,^[6]^ and the European Society for Medical Oncology guidelines recommend FDG-PET/CT for patients scheduled to undergo esophagectomy.^[8]^ The role of FDG-PET/CT in superficial esophageal cancer is limited and has not been sufficiently studied. The Japan Esophageal Society recommends that FDG-PET/CT be performed for all superficial esophageal cancers,^[7]^ whereas the European Society of Gastrointestinal Endoscopy does not recommend routine FDG-PET/CT before endoscopic resection.^[11]^ Little et al reported that FDG-PET/CT had no role in detecting superficial esophageal cancer.^[19]^ However, Miyata et al and Furukawa et al report that FDG-PET/CT is useful for detecting SM2 and SM3 cancers and predicting lymph node metastasis of thoracic superficial esophageal cancer.^[20,21]^ Both studies were limited because the number of patients was small (approximately 40). The sensitivity and specificity of FDG-PET/CT for deep SM cancer in the 2 aforementioned studies were 0.68 and 0.42 and 0.85 and 0.94, respectively.^[20,21]^ The results of our study showed that the sensitivity and specificity were 0.81 and 0.91, similar to the results of the 2 aforementioned studies. The sensitivity and specificity of PET for the detection of cancer with lymph node metastasis were 0.77 and 0.50 and 0.61 and 0.79, respectively.^[20,21]^ Our study results were similar (sensitivity, 0.78; specificity, 0.69). We used a fixed SUVmax cutoff of 2.5 because this threshold has been widely adopted in previous FDG-PET studies and has been routinely used in our institution for clinical interpretation.^[17]^

Currently, EUS and magnifying endoscopy with narrow-band imaging (ME-NBI) are the most widely recognized tools for predicting the depth of superficial esophageal cancer.^[22-24]^ As the T stage of esophageal cancer increases, the overall sensitivity of EUS also increases. During 2 meta-analyses, the sensitivity of EUS for T1 and T4 was 0.81 and 0.77 and 0.92 and 0.84, respectively; additionally, the specificity of EUS for T1 and T4 was 0.99 and 0.95 and 0.97 and 0.96, respectively.^[25,26]^ One meta-analysis reported sensitivity of 0.85 and specificity of 0.87 for differentiating T1a and T1b.^[24]^ However, the results of each study varied from 0.50 to 0.97 for sensitivity and 0.48 to 1.00 for specificity, indicating that the accuracy of EUS varies greatly depending on the institution and endoscopist. During our study, the sensitivity and specificity of EUS for T1b were 0.71 and 0.71, respectively. ME-NBI is also an excellent tool that can predict the invasion depth of superficial esophageal cancer, and both the sensitivity and specificity were 0.90 in the meta-analysis.^[27]^ Additionally, some studies that predicted the depth of invasion using ME-NBI plus EUS and the meta-analysis showed sensitivity of 0.79 and specificity of 0.94.^[23]^ One meta-analysis concluded that ME-NBI is a reliable modality for confirming deep cancer invasion, whereas EUS is a reliable modality for confirming mucosal cancer.^[22]^ However, ME-NBI was performed for only some patients and could not be analyzed during our study.

A prerequisite for endoscopic resection of superficial esophageal cancer is the absence of lymph node metastasis.^[7,11]^ Therefore, prediction of lymph node metastasis is most important before endoscopic resection or surgery; however, a test that can accurately predict lymph node metastasis is not available. One meta-analysis evaluated the detection of lymph node metastasis with esophageal cancer using EUS (47 articles), CT (25 articles), and FDG-PET/CT (12 articles), and the pooled sensitivity and specificity of EUS, CT, and FDG-PET/CT for regional lymph node metastasis were 0.764 and 0.724, 0.517 and 0.803, and 0.587 and 0.807, respectively.^[28]^ No studies have analyzed lymph node metastasis using each modality alone for T1 ESCC. One study analyzed the staging performance of EUS and FDG-PET/CT for T1 and T2 ESCC and showed that the sensitivity and specificity for differentiating node-positive disease were 0.90 and 0.75, respectively, for EUS and 0.89 and 0.39, respectively, for FDG-PET/CT.^[29]^ In our study, the sensitivity and specificity for differentiating node-positive disease were 0.97 and 0.29, respectively, for EUS and 0.98 and 0.44, respectively, for PET/CT. However, because only 9 patients had pathologically confirmed lymph node metastases, these diagnostic performance estimates are associated with wide CIs and should be interpreted with caution. Therefore, reliable conclusions regarding the role of FDG-PET/CT in nodal assessment cannot be drawn from the present study.

Superficial esophageal cancer involving the entire circumference is not a contraindication to endoscopic resection; however, strictures occur in 100% of cases after endoscopic resection if preventive measures are not performed.^[7]^ Even with oral or local injections of preventive steroids, the incidence of strictures remains high, and a large number of dilatations are required.^[7,30]^ Furthermore, 1 study reported that among T1a-M3/T1b-SM1 lesions, circumferential lesions had an ESD curability rate <20%.^[31]^ Therefore, if the lesion is a T1a-M3 whole circumferential lesion, even in the case of mucosal cancer, surgery or chemoradiotherapy, rather than endoscopic resection, is recommended by the treatment guidelines for esophageal cancer.^[7,11]^ In our study, mucosal cancers tended not to show FDG uptake in FDG-PET/CT; however, FDG uptake was observed in all whole or near-circumferential cancers, even mucosal cancers. Whole or near-circumferential cancer is thought to exhibit FDG uptake because of its large surface area. Although whole circumferential lesions can usually be identified by conventional endoscopy, FDG uptake may indicate a larger tumor burden. In our study, 5 of the 8 PET-positive mucosal cancers were whole circumferential lesions, suggesting that the additional diagnostic value of FDG-PET/CT beyond conventional endoscopy may be limited in these cases. Nevertheless, PET positivity alone should not be used to determine the treatment strategy and should be interpreted in conjunction with endoscopic findings.

FDG-PET/CT can incidentally detect other malignancies. Smoking and alcohol consumption are the most important causes of ESCC, and they can cause other cancers.^[32,33]^ The incidence of other cancers in patients with esophageal cancer ranges from 8.3% to 27.1%.^[33]^ Using FDG-PET/CT, synchronous neoplasms were found in 5.3% of patients with superficial esophageal cancer in 1 study.^[19]^ Using FDG-PET/CT during our study, other cancers were incidentally discovered in 3 patients (2.3%), and all were cured with surgery.

This study had some limitations. This was a single-center retrospective study with a relatively small number of patients. Second, test results can vary depending on the interpreter, and the accuracy of EUS, in particular, can vary depending on the operator’s experience. Furthermore, because EUS was unavailable in a subset of patients for nonrandom clinical reasons, selection bias may have influenced the estimated diagnostic performance of EUS. In addition, because all FDG-PET/CT images were interpreted by a single experienced nuclear medicine specialist, interobserver variability could not be evaluated. Third, because we only included patients with T1 esophageal cancer that was pathologically confirmed by surgery or endoscopic resection, some patients who underwent chemoradiotherapy were excluded. In addition, pathological confirmation of lymph node status was available only for surgically treated patients. Patients treated with endoscopic resection were considered node-negative based on pretreatment staging and surveillance follow-up rather than pathological assessment, which may have resulted in underestimation of occult lymph node metastasis. Fourth, several tumor characteristics, including tumor length, macroscopic type, lympho-vascular invasion, and invasion depth, were closely interrelated. Although exploratory multivariable analyses were performed, the models were unstable because of the limited number of outcome events and yielded unreliable estimates. Therefore, these analyses were not considered appropriate for presentation, and residual confounding cannot be excluded. Finally, partial-volume effects inherent to FDG-PET/CT may have reduced FDG uptake in small or flat superficial lesions, particularly type IIb cancers. Therefore, the observed association between FDG uptake and invasion depth may have been influenced, at least in part, by technical limitations of PET/CT.

## 5. Conclusions

FDG-PET/CT may be useful for differentiating submucosal invasive cancer and identifying whole or near circumferential lesions in T1 ESCC. FDG uptake on preoperative PET/CT may provide complementary information for risk stratification when selecting an appropriate treatment strategy. However, these findings should be considered hypothesis-generating and require validation in prospective multicenter studies using standardized FDG-PET/CT protocols and independent image interpretation before clinical implementation.

## Author contributions

**Conceptualization:** Dae Gon Ryu, Cheol Woong Choi.

**Data curation:** Seong Jang Kim, Su Jin Kim, Su Bum Park, Bong Soo Son.

**Formal analysis:** Jin Ook Jang.

**Funding acquisition:** Dae Gon Ryu.

**Investigation:** Dae Gon Ryu, Cheol Min Lee.

**Methodology:** Dae Gon Ryu, Cheol Woong Choi.

**Writing – original draft:** Dae Gon Ryu.

**Writing – review & editing:** Dae Gon Ryu, Cheol Woong Choi.

## References

[1] Eisner DC. Esophageal cancer: treatment advances and need for screening. JAAPA. 2024;37:19–24. 10.1097/01.JAA.0001007328.84376.da 38484297

[2] Rustgi AK El-Serag HB. Esophageal carcinoma. N Engl J Med. 2014;371:2499–509. 10.1056/NEJMra1314530 25539106

[3] Liu CQ Ma YL Qin Q. Epidemiology of esophageal cancer in 2020 and projections to 2030 and 2040. Thorac Cancer. 2023;14:3–11. 10.1111/1759-7714.14745 PMC9807450 36482832

[4] Ishihara R. Endoscopic diagnosis and treatment of superficial esophageal squamous cell cancer: present status and future perspectives. Curr Oncol. 2022;29:534–43. 10.3390/curroncol29020048 PMC8870955 35200548

[5] Shin CM. Treatment of superficial esophageal cancer: an update. Korean J Gastroenterol. 2021;78:313–9. 10.4166/kjg.2021.155 PMC12286475 34955507

[6] Ajani JA D’Amico TA Bentrem DJ. Esophageal and esophagogastric junction cancers, Version 2.2023, NCCN clinical practice guidelines in oncology. J Natl Compr Canc Netw. 2023;21:393–422. 10.6004/jnccn.2023.0019 37015332

[7] Kitagawa Y Ishihara R Ishikawa H. Esophageal cancer practice guidelines 2022 edited by the Japan esophageal society: part 1. Esophagus. 2023;20:343–72. 10.1007/s10388-023-00993-2 PMC10024303 36933136

[8] Obermannova R Alsina M Cervantes A; ESMO Guidelines Committee. Electronic address: clinicalguidelines@esmo.org. Oesophageal cancer: ESMO clinical practice guideline for diagnosis, treatment and follow-up. Ann Oncol. 2022;33:992–1004. 10.1016/j.annonc.2022.07.003 35914638

[9] Yamashina T Ishihara R Nagai K. Long-term outcome and metastatic risk after endoscopic resection of superficial esophageal squamous cell carcinoma. Am J Gastroenterol. 2013;108:544–51. 10.1038/ajg.2013.8 23399555

[10] Akutsu Y Uesato M Shuto K. The overall prevalence of metastasis in T1 esophageal squamous cell carcinoma: a retrospective analysis of 295 patients. Ann Surg. 2013;257:1032–8. 10.1097/SLA.0b013e31827017fc 23108117

[11] Pimentel-Nunes P Libanio D Bastiaansen BAJ. Endoscopic submucosal dissection for superficial gastrointestinal lesions: European Society of Gastrointestinal Endoscopy (ESGE) Guideline - Update 2022. Endoscopy. 2022;54:591–622. 10.1055/a-1811-7025 35523224

[12] Kitajima K Nakajo M Kaida H. Present and future roles of FDG-PET/CT imaging in the management of gastrointestinal cancer: an update. Nagoya J Med Sci. 2017;79:527–43. 10.18999/nagjms.79.4.527 PMC5719212 29238109

[13] Himeno S Yasuda S Shimada H Tajima T Makuuchi H. Evaluation of esophageal cancer by positron emission tomography. Jpn J Clin Oncol. 2002;32:340–6. 10.1093/jjco/hyf073 12417599

[14] Kamel EM Thumshirn M Truninger K. Significance of incidental 18F-FDG accumulations in the gastrointestinal tract in PET/CT: correlation with endoscopic and histopathologic results. J Nucl Med. 2004;45:1804–10. 15534047

[15] Japan Esophageal Society. Japanese classification of esophageal cancer, 11th Edition: part I. Esophagus. 2017;14:1–36. 10.1007/s10388-016-0551-7 PMC5222932 28111535

[16] Catalano MF Sivak MV Jr Rice T Gragg LA Van Dam J. Endosonographic features predictive of lymph node metastasis. Gastrointest Endosc. 1994;40:442–6. 10.1016/s0016-5107(94)70206-3 7926534

[17] Shum WY Hsieh TC Yeh JJ. Clinical usefulness of dual-time FDG PET-CT in assessment of esophageal squamous cell carcinoma. Eur J Radiol. 2012;81:1024–8. 10.1016/j.ejrad.2011.03.018 21458941

[18] Shweikeh F Zeng Y Jabir AR. The emerging role of blood-based biomarkers in early detection of colorectal cancer: a systematic review. Cancer Treat Res Commun. 2024;42:100872. 10.1016/j.ctarc.2025.100872 39892077

[19] Little SG Rice TW Bybel B. Is FDG-PET indicated for superficial esophageal cancer? Eur J Cardiothorac Surg. 2007;31:791–6. 10.1016/j.ejcts.2007.01.037 17337344

[20] Miyata H Doki Y Yasuda T. Evaluation of clinical significance of 18F-fluorodeoxyglucose positron emission tomography in superficial squamous cell carcinomas of the thoracic esophagus. Dis Esophagus. 2008;21:144–50. 10.1111/j.1442-2050.2007.00743.x 18269650

[21] Furukawa T Hamai Y Hihara J. Clinical significance of FDG-PET to predict pathologic tumor invasion and lymph node metastasis of superficial esophageal squamous cell carcinoma. Ann Surg Oncol. 2016;23:4086–92. 10.1245/s10434-016-5359-0 27352201

[22] Ishihara R Matsuura N Hanaoka N. Endoscopic imaging modalities for diagnosing invasion depth of superficial esophageal squamous cell carcinoma: a systematic review and meta-analysis. BMC Gastroenterol. 2017;17:24. 10.1186/s12876-017-0574-0 PMC5288972 28152974

[23] Su F Zhu M Feng R Li Y. ME-NBI combined with endoscopic ultrasonography for diagnosing and staging the invasion depth of early esophageal cancer: a diagnostic meta-analysis. World J Surg Oncol. 2022;20:343. 10.1186/s12957-022-02809-6 PMC9575268 36253783

[24] Thosani N Singh H Kapadia A. Diagnostic accuracy of EUS in differentiating mucosal versus submucosal invasion of superficial esophageal cancers: a systematic review and meta-analysis. Gastrointest Endosc. 2012;75:242–53. 10.1016/j.gie.2011.09.016 22115605

[25] Puli SR Reddy JB Bechtold ML Antillon D Ibdah JA Antillon MR. Staging accuracy of esophageal cancer by endoscopic ultrasound: a meta-analysis and systematic review. World J Gastroenterol. 2008;14:1479–90. 10.3748/wjg.14.1479 PMC2693739 18330935

[26] Luo LN He LJ Gao XY. Endoscopic ultrasound for preoperative esophageal squamous cell carcinoma: a meta-analysis. PLoS One. 2016;11:e0158373. 10.1371/journal.pone.0158373 PMC4936717 27387830

[27] Yu T Geng J Song W Jiang Z. Diagnostic accuracy of magnifying endoscopy with narrow band imaging and its diagnostic value for invasion depth staging in esophageal squamous cell carcinoma: a systematic review and meta-analysis. Biomed Res Int. 2018;2018:8591387. 10.1155/2018/8591387 PMC5985084 29888281

[28] Sgourakis G Gockel I Lyros O Hansen T Mildenberger P Lang H. Detection of lymph node metastases in esophageal cancer. Expert Rev Anticancer Ther. 2011;11:601–12. 10.1586/era.10.150 21504265

[29] Jeong DY Kim MY Lee KS. Surgically resected T1- and T2-stage esophageal squamous cell carcinoma: T and N staging performance of EUS and PET/CT. Cancer Med. 2018;7:3561–70. 10.1002/cam4.1617 PMC6089181 29932307

[30] Sato H Inoue H Kobayashi Y. Control of severe strictures after circumferential endoscopic submucosal dissection for esophageal carcinoma: oral steroid therapy with balloon dilation or balloon dilation alone. Gastrointest Endosc. 2013;78:250–7. 10.1016/j.gie.2013.01.008 23453294

[31] Matsueda K Matsuura N Kanesaka T. Validity of endoscopic resection for clinically diagnosed T1a-MM/T1b-SM1 N0 M0 esophageal squamous cell carcinoma. Esophagus. 2021;18:585–93. 10.1007/s10388-021-00814-4 33475874

[32] Cui Y Ren W Du X Yang L Tan B. Research progress of multiple primary malignancies associated with esophageal cancer. Cancer Control. 2023;30:10732748231176641. 10.1177/10732748231176641 PMC10214099 37212379

[33] Nandy N Dasanu CA. Incidence of second primary malignancies in patients with esophageal cancer: a comprehensive review. Curr Med Res Opin. 2013;29:1055–65. 10.1185/03007995.2013.816276 23777310

